# Supplementary material for: The neuropeptide F/nitric oxide pathway is essential for shaping locomotor plasticity underlying locust phase transition
Source: eLife. 2017 Mar 27;6:e22526. doi: 10.7554/eLife.22526 (PMC5400507; doi:10.7554/eLife.22526)
Supplement: Supplementary file 1. — DOI: http://dx.doi.org/10.7554/eLife.22526.034 [file elife-22526-supp1.docx]

**Supplement file 1. Protein sequences of two NPF receptors, NPFR and NPYR, in the migratory locust.**

*Locusta* NPFR

MAEVPGLGAGAAALLPTAVPSLPGVARNLSVHITRILNSSQVNPHIFDQYSRNRKLDDSAAFYGLIAAYSLLILIGAAGNSLVVCAVARKPAMRTARNMFIVNLAVSDLLLCLVTMPLTLMEILTKYWPLGRQVFICKMLGALQATSIFVSTISITAIALDRYQVIVYPTRESLQKVGAIVILGCIWLVSLVLASPMFIWRALKNHDINLPDLPYISYCLEDWPVEHGRLYYSVFSLVVQYLLPIITVTVAYSRICHKLRYRYVNSSIANGKGGGGDAKASCSKHTSVRRKPKDDRRMKRTNSLLYSIALIFCISWLPLNIFNLVMDLWNPPVHDEQTMIICYAVCHMMGMSSACSNPLLYGWLNDNFRKEFHEIAAAVCPCVKLEAVRERVGSLRSSMTRRRHRRQEEADSKAATGAAGASTGRTSSRRSEAGGGGGGGAGAPARRARETPPGEGASPPREGDAAAAQADGARLRGRRHQHKESALGTTTY

*Locusta* NPYR

MGSSSEMVTVDDDVLTNSTTNICEMPGSTALSSTPFVALVVVLYGVILLGSVVGNGLVVYTITRNRAMRTVTNLLLLNLALGDLLLTLVCIPFVALPVLILQHWPFGQLLCQLVSCVQGIGVLVSAFTLIAVSGDRYWALRWPLRVRLGVRGAQRLMLFIWLLAAVTAAPIGVVSRLSQPTEWHVRCDRFMCREEWQNENHRYIYTLTLLSLQYLVPVTCLFVTYVLIVRMVWNRPQPGAGYYLRETRAVQSRRKVIVMMIAVVTAFLICWLPLNVLLLVAEHRPALEQWEGLPYLYLFCHWFAMSHACCNPVIYSWLNARFRAGFCLGLSKMPVIGKWIPHHTILDAVELVGGLSRRPSLRPELASHQRLRMPFRRPTSASSATAYGGDSPFFAETSFNFPEPQQLVSAALRRSRSVIGGGDAGSSADRPRARSVGRVLVDHIRRPSPAPADDQDSDIAANLTPPARPLLNRQSSVPHQRSFFQVTGRESARLQRPRTNLGAGRGAEGRVSPSAPPRGGGPRLTLNSLASGLGDAIDTFRQEMELQKRRVARESRRRERIEAEMRAMAGPSGAQPAQCVAKRYWPSRRPTPPRKAASQPPARPPPRPSTPPPPPPPPPPEDAERRSCCSLTMCFRGSPED-
