# Supplementary material for: The neuropeptide F/nitric oxide pathway is essential for shaping locomotor plasticity underlying locust phase transition
Source: eLife. 2017 Mar 27;6:e22526. doi: 10.7554/eLife.22526 (PMC5400507; doi:10.7554/eLife.22526)
Supplement: Supplementary file 2. — DOI: http://dx.doi.org/10.7554/eLife.22526.035 [file elife-22526-supp2.docx]

**Supplementary file 2.** Primers used in qPCR and RNAi expriments.

| **Gene name** | **Forward primer**  **(5’-3’)** | **Reverse primer**  **(5’-3’)** |
| --- | --- | --- |
| **ACP RT** | GCAAGGCTGCTCCTCACA | CTTCCACCATTTCCATCAA |
| **ACPRNAi** | TAATACGACTCACTATAGG  GCAAGGCTGCTCCTCACA | TAATACGACTCACTATAGG  CTTCCACCATTTCCATCAA |
| **AT** | CGCACGATACGCGGCTTCA | AGTGGGCCTGAGAAGCTC |
| **DH-Calc** | ACTACGTGTCAGACTACG | CAGCCCCATCAGGTGCTT |
| **ILP RT** | ATGATGTGGAAGCTGTGCCT | GCGGCGGCCGCAGTAGGTCT |
| **ILP RNAi** | TAATACGACTCACTATAGG  ATGATGTGGAAGCTGTGCCT | TAATACGACTCACTATAGG  CTCCGAGTCTGACACATCT |
| **ITP-A** | CAGTGGCGGTTATCAGGG | GTAGTGCCTGAAGACAACC |
| **MS1** | ATCCGCAAGGTGTGCCAAG | GAAGACGTGGTCCACATCG |
| **NPP2** | TCCTCGCTGTCACCCTGTT | TTCTGGTATCAGATCACCG |
| **NPP4** | TCCTCGCTGTCACCCTGTT | TGTCGAAGGCCTAGGGACA |
| **NPP5** | TCCTCGCTGTCACCCTGTT | TGCCGTGCAGCTCGTAG |
| **NPF1a RT** | GCGCGCCCAGAGCGGCC | GAACCTGGGCCGGCCGAG |
| **NPF1a RNAi** | TAATACGACTCACTATAGG  GCTACTACTCGCAGGTGG | TAATACGACTCACTATAGG  CGGATGTCGTCGATGACG |
| **NPF2 RT** | CGGCAACAAGCTGGAGG | CAGAACTTGTCAGCGCTC |
| **NPF2 RNAi** | TAATACGACTCACTATAGG  GCTGTCTGCGCCGGACGTG | TAATACGACTCACTATAGG  GAACCTGGGCCGGCCGAG |
| **OK-A** | CGTGGAGGAAGGATTGGTG | GAGTCCGCTCTTGGTCTGC |
| **PDF** | TGTCCTTGGTCTGGCACT | CTGAGCGAGCAATGTAG |
| **sNPF** | ATGGCATCGACGTCCGCC | CACGGCGCTTTCCGGAGC |
| **TK** | CAAGAAGGGGCCCTCCGGCT | GAAGCCGACGGGCGCCTTCT |
| **NOS RT** | CGAGACATTCTGCCTTGA | ACGGCTGGCATCATTTCC |
| **NOS RNAi** | TAATACGACTCACTATAGG  GAAGACTGGAGGCATTG | TAATACGACTCACTATAGG  CGTGAAAGGGCAAGGA |
| **LOCMI3391** | GAGTTTGATGAGGCGGAGCA | CGTGCCATGTTGATTGACC |
| **LOCMI6792** | GCCACCTTATTCCTTCTCC | TTGGGTCAACCATCCACAGC |
| **ti** | CGAGGAGAACATTGCCATCA | ATCGGGAACTCGGTCTGC |
| **LOCMI8397** | TCTACCTGCCCTGCCACTA | GAATCGGTACTGACCATC |
| **LOCMI9067** | GCGATGTTTACAGGTGG | TCCCACTGCGTAGAACA |
| **RP49** | CGTAAACCGAAGGGAATTGA | GAAGAAACTGCATGGGCAAT |
| **NPFR RT** | GCTCAAGAACCACGACATCAAC | CCTTGCGGAAGTTGTCGTTG |
| **NPFR RNAi** | TAATACGACTCACTATAGG TGGCTGGTGTCGCTGGTGCT | TAATACGACTCACTATAGG CTCGTGGAACTCCTTGCGA |
| **NPYR RT** | CTGCCCGTGCTCATCCTGC | CAGAGGCAACCAGCAAATC |
| **NPYR RNAi** | TAATACGACTCACTATAGG ATGGGAAGCTCAAGCGAGAT | TAATACGACTCACTATAGG GGTCACACCTGACGTGCCAC |
| **NPFR OvE** | TATCGGTACCGCCATGGCGGAGGTGCCGGGG | TATCGAATTCCTACTTATCGTCGTCATCCTTGTAATCATACGTTGTCGTTCC |
| **NPYR OvE** | TATCGGTACCGCCATGGGAAGCTCAAGCGAG | TATCGAATTCCTACTTATCGTCGTCATCCTTGTAATCATCCTCTGGAGAGCC |

Red font indicates T7 promoter sequence.
